# Targeting enhancer of zeste homolog 2 protects against acute kidney injury

**DOI:** 10.1038/s41419-018-1012-0

**Published:** 2018-10-19

**Authors:** X. Zhou, X. Zang, Y. Guan, T. Tolbert, T. C. Zhao, G. Bayliss, S. Zhuang

**Affiliations:** 1 Department of Medicine, Rhode Island Hospital and Alpert Medical School, Brown University, Providence, RI 02903 USA; 2 Departments of Cardiology, First Affiliated Hospital of Harbin Medical University, 150001 Harbin, Heilongjiang Province China; 3 Department of Nephrology, Shanghai Songjiang District Central Hospital, 201600 Shanghai, China; 4 Department of Surgery, Boston University Medical School, Roger Williams Medical Center, Boston University, Providence, RI 02908 USA; 5 Department of Nephrology, Shanghai East Hospital, Tongji University School of Medicine, 200120 Shanghai, China

## Abstract

Despite the established oncogenic and profibrotic functions of enhancer of zeste homolog 2 (EZH2), a methyltransferase that induces histone H3 lysine 27 trimethylation (H3K27me3), its role in acute kidney injury (AKI) remains unclear. In this study, we demonstrated that EZH2 and H3K27me3 were upregulated in the murine kidney with AKI induced by either ischemia-reperfusion (I/R) or folic acid (FA). Pharmacologic inhibition of EZH2 with 3-deazaneplanocin A (3-DZNeP) prevented tubular injury in both models as demonstrated by reduced renal dysfunction, diminished neutrophil gelatinase-associated lipocalin expression and decreased renal tubular cell death. Injury to the kidney resulted in reduced expression of E-cadherin and ZO-1, whereas EZH2 inhibition largely preserved their expression. Moreover, 3-DZNep was effective in counteracting the increased expression of matrix metalloproteinase (MMP)-2 and MMP-9, as well as the phosphorylation of Raf-1 and ERK1/2 in the injured kidney. Conversely, blocking EZH2 reversed the decrease of tissue inhibitor of metalloproteinase (TIMP)-2 and metalloproteinase (TIMP)-3, and Raf kinase inhibitor protein (RKIP) in the kidney after acute injury. Similarly, oxidant injury to cultured kidney proximal tubular epithelial cells caused a decrease in the expression of E-cadherin, ZO-1, TIMP-2/-3, and RKIP, as well as an increase in the expression of MMP-2/9 and phosphorylation of Raf-1 ERK1/2. Blocking EZH2 with 3-DZNep or SiRNA hindered these responses. Thus, these results suggest that targeting EZH2 protects against AKI through a mechanism associated with the preservation of adhesion/junctions, reduction of matrix metalloproteinases and attenuation of the Raf-1/ERK1/2 pathway.

## Introduction

Acute kidney injury (AKI) is a common clinical problem and occurs under a variety of pathological conditions^1,2^. To date, there are no effective treatments for AKI aside from supportive measures and renal replacement therapy. Thus, it is important to elucidate the mechanisms of AKI and develop novel approaches to treat this disease. Previously, studies have shown that renal tubular cell death is a key pathological process in AKI and is regulated by activation of cell death receptors and induction of mitochondrial damage^3,4^. Disruption of E-cadherin-mediated cell–cell adhesion^5^ and activation of matrix metalloproteinases (MMPs)^6^ and extracellular signal-regulated kinase-1/2 (ERK1/2) are also reported to contribute to this process^7,8^. Recently, studies have also demonstrated that occurrence of AKI is associated with epigenetic regulation, such as acetylation and changes in microRNA expression levels^9^. The role of histone methylation in AKI, however, remains poorly understood.

Protein methylation is one of the major histone modifications and is catalyzed by histone methyltransferases and offset by histone demethylases^10^. Histone methylation can either increase or decrease transcription of gene, depending on which amino acids of histones are methylated^11^. The protein methyltransferase enhancer of zeste homolog 2 (EZH2) methylates lysine 27 of histone H3 (H3K27) to promote transcriptional silencing^12^ and can induce methylation of both histone and non-histone proteins^13^. A growing body of evidence indicates that EZH2 is linked to the expression and/or activation of multiple genes and signaling molecules involving various cellular responses, including apoptosis^14,15^. For example, EZH2 activity is required for the suppression of genes associated with cell death, such as E-cadherin, tissue inhibitor 3 of metalloproteinases (TIMP3) and Raf-1 kinase inhibitor protein (RKIP)^16–18^. E-cadherin can also act together with tight junction proteins like ZO-1 to form adherent/tight junctions, which are necessary for the establishment of renal epithelial polarity and cell survival^19^. TIMP3 is one of a family of proteins with the ability to inhibit MMP, such as MMP-2 and MMP-9^20,21^. Downregulation and/or inactivation of TIMP3 can induce and/or increase MMP-2 and MMP-9 activities. MMP-2 and MMP-9 activities are increased in the renal tubules of rat kidneys after IR and involved in the pathogenesis of ischemic AKI^22,23^. As a suppressor of Raf-1, RKIP is able to block the signal transduction from Raf-1 to ERK1/2, thereby leading their activation^24^. Activation of ERK1/2 has been shown to contribute to renal epithelial cell death in the kidney after acute injury^25,26^.

The present study was undertaken to examine the role of EZH2 in murine models of AKI induced by ischemia/reperfusion (I/R) and folic acid (FA) and cultured renal epithelial cells and to uncover the mechanisms involved. We found that pharmacological inhibition of EZH2 with 3-deazaneplanocin A (3-DZNeP), a selective inhibitor of EZH2, or siRNA-mediated silencing of EZH2, protects against AKI and alleviates renal tubular cell death in the murine kidney of I/R or FA-induced AKI. EZH2 siRNA also inhibited renal tubular cell death in vitro.

## Results

### Blocking EZH2 with 3-DZNep protects against AKI induced by I/R and FA in mice

**Fig. 1:**
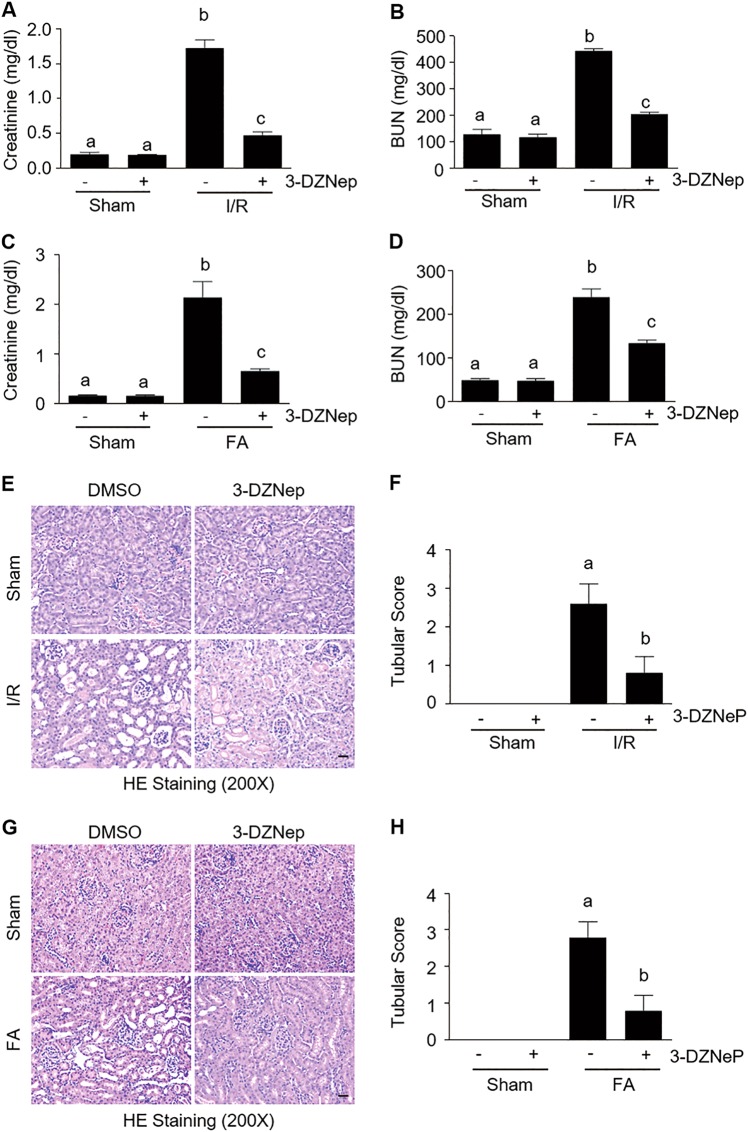
EZH2 inhibition protects against AKI induced by ischemia-reperfusion (I/R) and folic acid (FA) in mice. After various treatments as indicated, blood was collected and the serum creatinine and blood urea nitrogen (BUN) were measured (**a**–**d**). The kidneys injured by IR (**e**) and FA (**g**) underwent hematoxylin and eosin (HE) staining. Scale bar, 20 µm. Morphological changes induced by IR (**f**) and FA (**h**) were scored based on the scale described in Material and methods. Data are represented as means ± SD (*n* = 3). Means with different superscript letters are significantly different from one another (*P* < 0.05)

To assess whether EZH2 is implicated in AKI, we first examined the effects of 3-DZNep, an inhibitor of S-adenosylmethionine-dependent methyltransferase that targets the degradation of EZH2^27^, on renal function in murine models of IR or FA. 3-DZNep was administrated immediately after the onset of reperfusion or after FA injection and then administered daily for two consecutive days. The blood and kidney tissue were collected at 24 or 48 h after the treatment. As shown in Fig. 1a, b, the serum creatinine and BUN levels were increased in IR-injured mice, while administration of 3-DZNep significantly reduced their levels. Similarly, 3-DZNep treatment also significantly suppressed the rise in serum creatinine and BUN levels in mice with FA administration (Fig. 1c, d). Kidney damage was characterized by the presence of tubular dilatation, swelling, necrosis, and/or luminal congestion. Renal injury was clearly observed in the kidneys of mice subjected to IR or FA, and treatment with 3-DZNep clearly attenuated these effects (Fig. 1e, g). Scoring of kidney sections showed less severe tubular damage in the injured kidneys after 3-DZNep administration (Fig. 1f, h). These data indicate that EZH2 inhibition protects against renal injury and lessens renal dysfunction in animal models of AKI induced by IR or FA.

### Administration of 3-DZNep decreases EZH2 expression levels and inhibits histone H3K27 trimethylation in the kidneys of mice with IR or FA-induced AKI

**Fig. 2:**
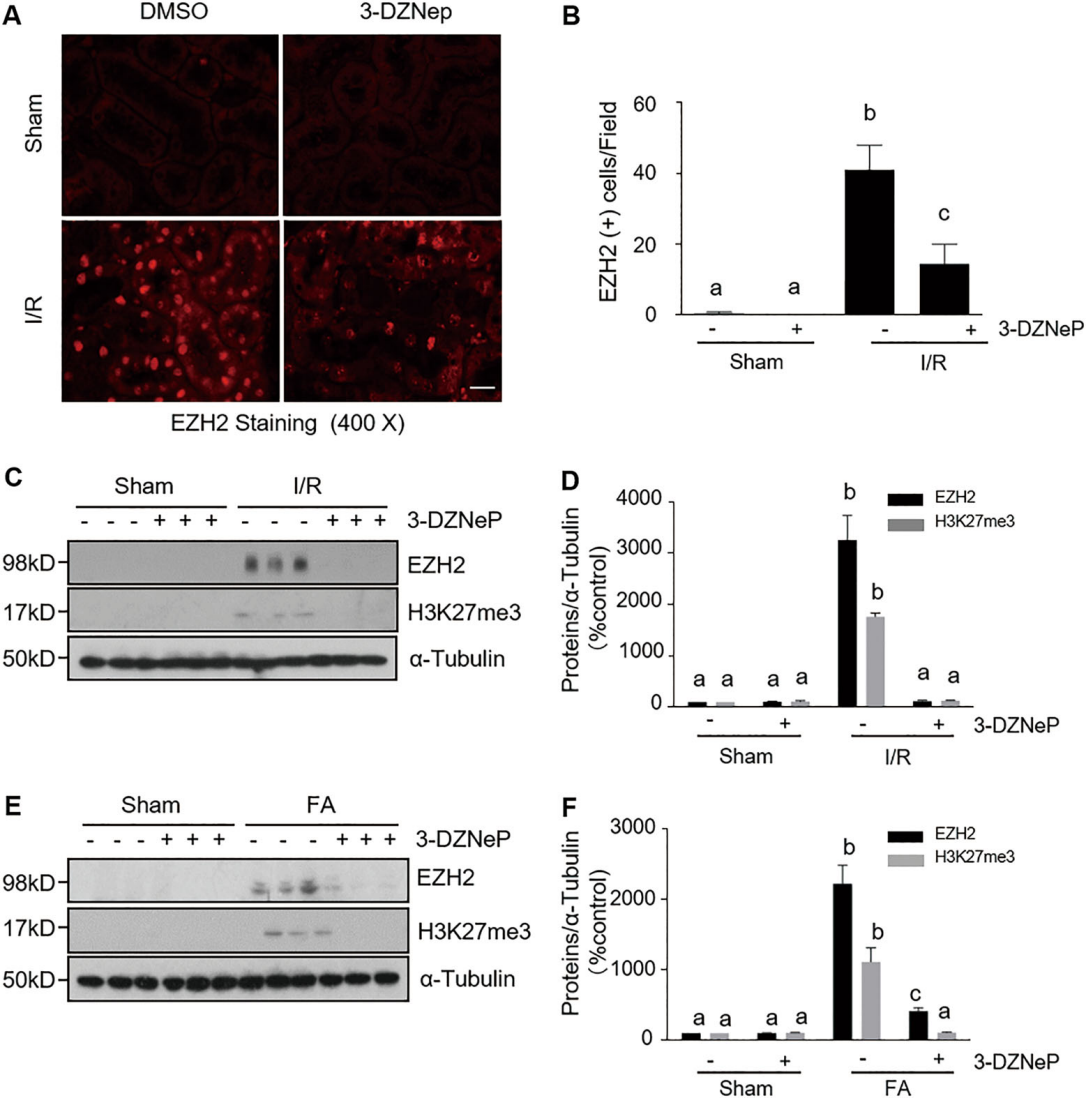
EZH2 inhibition blocks expressions of EZH2 and of H3K27me3 in the kidneys of IR-induced or FA-induced AKI. **a** Photomicrographs illustrate EZH2 with immunofluorescent staining of the kidney tissues collected at 48 h after IR injuring with or without 3-DZNep administration in C57/black mice. Scale bar, 20 µm. **b** Tubular cells with positive EZH2 staining were counted in 10 high-power fields and expressed as means ± SD. **c**, **e** Kidneys injured by IR or FA, respectively, tissue lysates were subjected to immunoblot analysis with specific antibodies against EZH2, H3K27me3, and ɑ-tubulin. **d**, **f** Expression levels of EZH2 and H3K27me3 were quantified by densitometry and normalized with ɑ-tubulin. Representative immunoblots are three samples in each group. Means with different superscript letters are significantly different from one another (*P* < 0.05)

To demonstrate the inhibitory effect of 3-DZNep on the expression and activation of EZH2 in the injured kidney, we examined expression levels of renal EZH2 and H3K27me3. As shown in Fig. 2a, b, immunofluorescence staining revealed very few EZH2-positive cells in the sham-operated kidney. By contrast, increased numbers of EZH2-positive cells were observed in the kidneys injured by IR. Treatment with 3-DZNep significantly reduced this population of cells in the injured kidney. Of note, EZH2 is most often found within the cell nucleus, and a minimal expression in the cytosol in renal tubule cells of the kidney after injury. To confirm this observation, we also examined the expression levels of EZH2 and H3K27me3 by immunoblot analysis. As shown in Fig. 2c–f, a minimal level of EZH2 was detected in the sham-operated kidney without 3-DZNep treatment. Expression levels of EZH2 and H3K27me3 were dramatically increased in kidneys injured by IR or FA. 3-DZNep treatment abolished H3K27me3 expression and largely reduced the expression of EZH2. Thus, 3-DZNep is effective in reducing expression of EZH2 and suppressing H3K27 trimethylation in the injured kidney.

### EZH2 inhibition with 3-DZNep attenuates renal tubular damage after IR or FA injury in mice

**Fig. 3:**
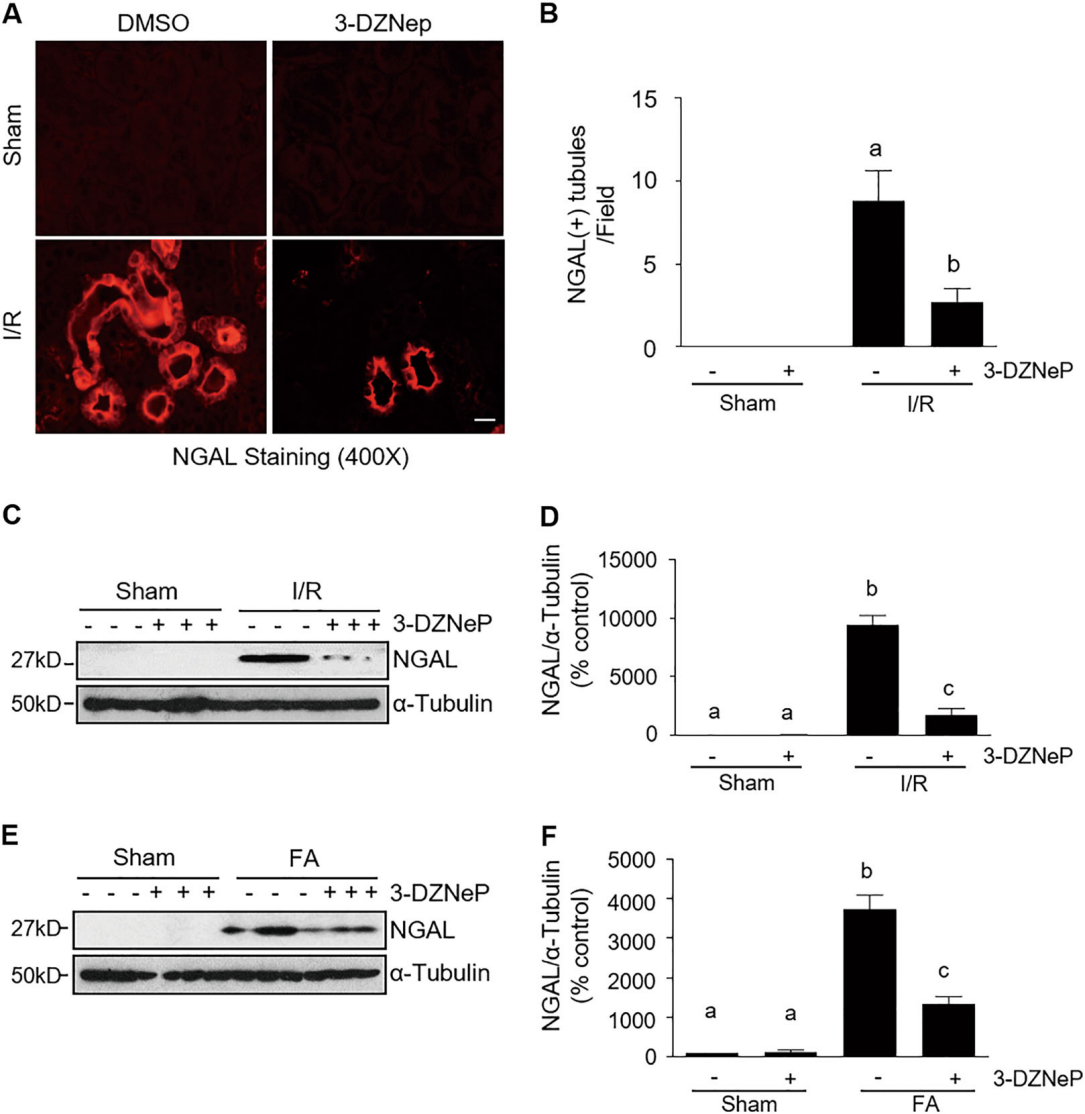
EZH2 inhibition decreases expression of neutrophil gelatinase-associated lipocalin (NGAL) in the kidney of IR-induced or FA-induced AKI. **a** Photomicrographs illustrate immunofluorescent staining of NGAL in kidney tissues collected at 48 h after sham and IR injury with or without 3-DZNep administration in C57/black mice. **b** Tubules with positive NGAL staining were counted in 10 high-power fields and expressed as means ± SD. Scale bar, 20 µm. **c**, **e** Kidneys were injured by IR or FA, respectively, and tissue lysates were subjected to immunoblot analysis with specific antibodies against NGAL and ɑ-tubulin. **d**, **f** Expression levels of NGAL were quantified by densitometry and normalized with ɑ-tubulin. Data are means ± SD. Representative immunoblots are three samples in each group

To determine the role of EZH2 in renal tubular cell damage, we examined expression of NGAL, a tubule injury marker, by immunofluorescence staining in the kidney of IR-induced AKI in mice. As expected, NGAL expression is limited to renal tubular cells. Although the NGAL signal was not observed in the sham-operated kidney, it was clearly seen in some tubules in the kidneys injured by IR; treatment with 3-DZNep inhibited this response (Fig. 3a, b). In agreement with this observation, NGAL was not detected in sham-operated kidneys by immunoblot analysis, but induced in the IR and FA injured kidneys; 3-DZNep treatment attenuated expression of NGAL (Fig. 3c, f). In addition, we observed that 3-DZNep inhibited renal NGAL expression early in the time course of AKI (24 h) after IR injury (Supplemental Fig. 1A-B). These data indicate that EZH2 inhibition protects against ischemic and toxic AKI.

### EZH2 inhibition attenuates renal tubular cell death in the murine model of IR-induced or FA-induced AKI and in cultured renal proximal tubular cells following oxidant injury

**Fig. 4:**
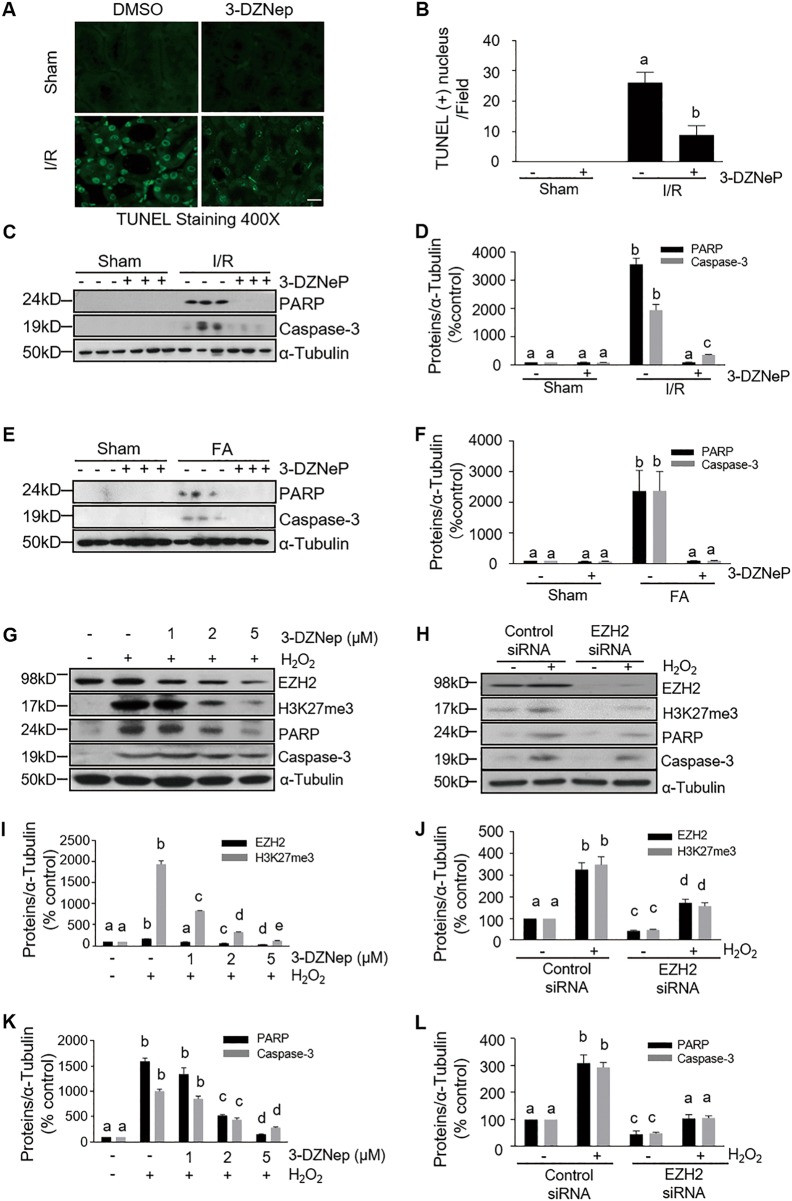
EZH2 inhibition attenuates renal tubular cell apoptosis in the kidney of IR-induced or FA-induced AKI and protects against apoptosis in H_2_O_2_-injured TKPT. **a** Photomicrographs illustrate TdT-mediated dUTP nick-end labeling (TUNEL) staining of the kidney tissues collected at 48 h after sham and IR injuring with or without 3-DZNep administration in C57/black mice. Scale bar, 20 µm. **b** Positive TUNEL staining cell nuclei were counted in 10 high-power fields and expressed as means ± SD. **c**, **e** Kidneys were injured by IR or FA, respectively, and tissue lysates were subjected to immunoblot analysis with specific antibodies against cleaved PARP (24 kDa), cleaved caspase-3 (17 kDa), and ɑ-tubulin. **d**, **f** Expression levels of cleaved PARP and cleaved caspase-3 were quantified by densitometry and normalized with ɑ-tubulin, respectively. Data are means ± SD. Representative immunoblots are three samples in each group. Cultured TKPT were pretreated with various concentration of 3-DZNep (1–5 μM) for 1 h (**g**, **i**, **k**) or transfected with control or EZH2 siRNA for 24 h (**h**, **j**, **l**) and then exposed to 0.5 mM H_2_O_2_ for an additional 12 h. Immunoblot analysis were conducted with specific antibodies against EZH2, H3K27me3, cleaved PARP (24 kDa), cleaved caspase-3 (17 kDa) and ɑ-tubulin (**g**, **h**); expression levels of EZH2, H3K27me3 (**i**, **j**), cleaved PARP (24 kDa) and cleaved caspase-3 (17 kDa) (**k**, **l**) were quantified by densitometry and normalized with ɑ-tubulin. Data are means ± SD from at least three individual experiments. Means with different superscript letters are significantly different from one another (*P* < 0.05)

Following AKI, most renal tubular cells die by apoptosis. To examine the effect of EZH2 inhibition on renal tubular cell death, we further examined the effect of 3-DZNep on apoptosis in the kidney with a TUNEL assay. TUNEL-positive cells were not observed in the sham-operated kidney, but were evident in the kidney injured by IR. 3-DZNep administration significantly reduced the number of apoptotic tubular cells in IR-injured kidney (Fig. 4a, b). To confirm the role of EZH2 in mediating apoptosis, we examined expression levels of two hallmarks of apoptosis by immunoblot analysis: cleaved caspase-3 at 17-kDa fragments and cleaved poly (ADP-ribose) polymerase (PARP) at 24-kDa fragments. As shown in Fig. 4c–f, IR-induced or FA-induced expression of both cleaved PARP and cleaved caspase-3 was largely inhibited by 3-DZNep administration. It should be noted that 3-DZNep itself did not induce cleavage of caspase-3 and PARP in the sham-operated kidney. These data illustrate that EZH2 contributes to apoptosis of renal tubular cells after acute injury.

To validate the above observation, we further examined the effect of EZH2 inhibition on the death of mouse proximal tubule epithelial (TKPT) cells following oxidant injury induced by H_2_O_2_. As shown in Supplemental Fig. 2A, B, exposure of TKPT cells to different concentrations of 3-DZNep (1–5 μM) or a single concentration of siRNA EZH2 (100 pM) protected against H_2_O_2_-indued death of TKPT cells as examined by the MTT assay. In parallel, 3-DZNp treatment dose-dependently suppressed cleavage of caspase-3 and PARP in cultured TKPT exposed to H_2_O_2_ (Fig. 4g, k). Furthermore, silencing of EZH2 with its specific siRNA inhibited H_2_O_2_-induced cleavage of caspase-3 and PARP (Fig. 4h, l). Notably, EZH2 and H3K27me3 protein levels were successfully downregulated in cells treated with 3-DZNep or EZH2 siRNA (Fig. 4g–j).

### Inhibition of EZH2 attenuates disruption of renal epithelial cell adherent/tight junctions in the kidney following IR and in cultured renal proximal tubular cells following oxidant injury

**Fig. 5:**
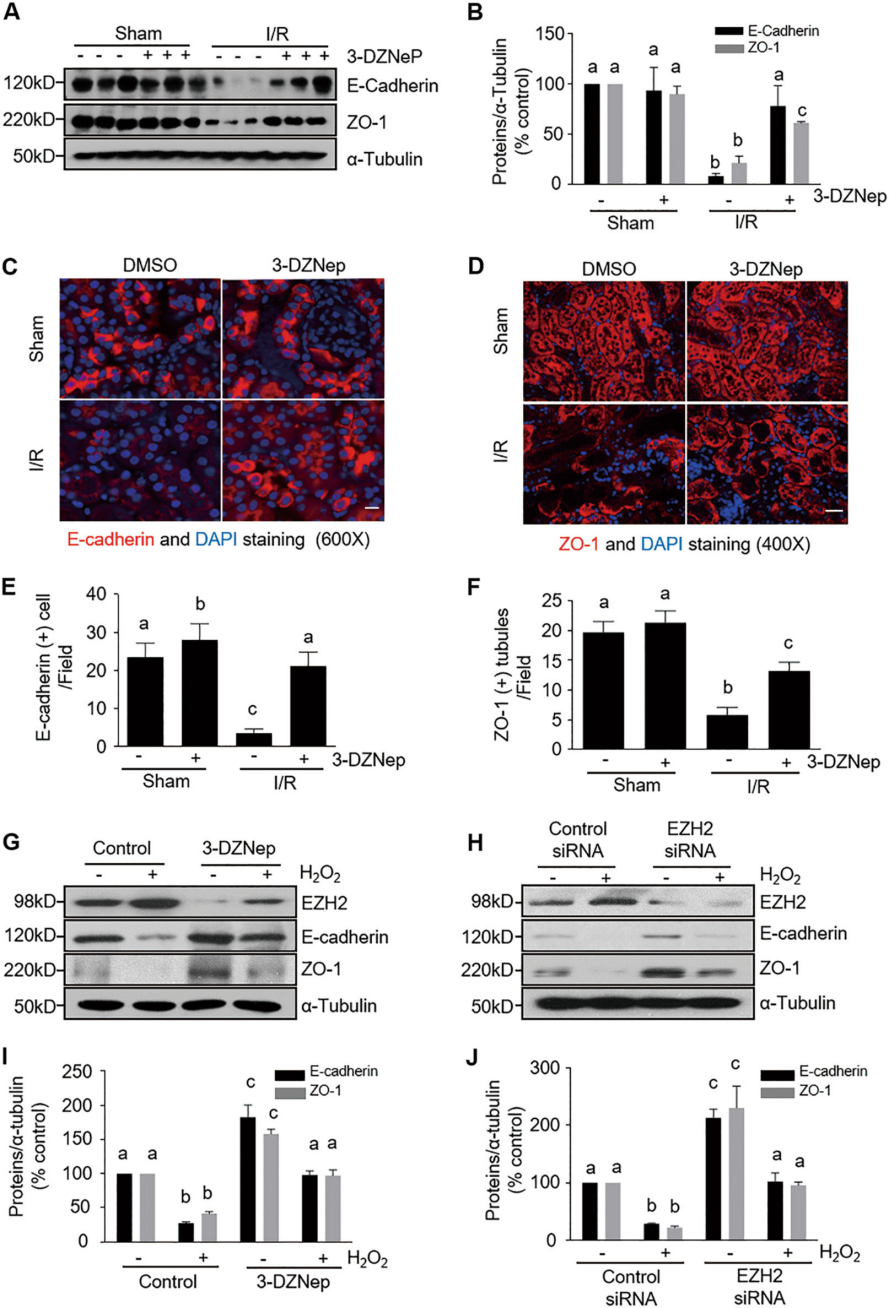
EZH2 inhibition ameliorates tubular cell adherence and junction damage in the kidney of IR-induced AKI and H_2_O_2_-injured TKPT. **a** Kidney tissue lysates were subjected to immunoblot analysis with specific antibodies against E-cadherin, ZO-1 and ɑ-tubulin. **b** Expression levels of E-cadherin and ZO-1 were quantified by densitometry and normalized with ɑ-tubulin. Data are means ± SD. Representative immunoblots are three samples from in each group. Photomicrographs illustrate immunofluorescent staining of E-cadherin (**c**) (scale bar, 10 µm) or ZO-1 (**d**) (scale bar, 20 µm) in kidney tissues collected at 48 h after sham and IR injuring with or without 3-DZNep administration in C57/black mice. Tubular cells with positive E-cadherin (**e**) or ZO-1 (**f**) staining were counted in 10 high-power fields and expressed as means ± SD. TKPT were pretreated with 5 µM 3-DZNep for 1 h or transfected with control or EZH2 siRNA for 24 h and then exposed to 0.5 mM H_2_O_2_ for an additional 12 h. Cell lysates were subjected to immunoblot analysis with specific antibodies against EZH2, E-cadherin, ZO-1, and ɑ-tubulin (**g**, **h**) and expression levels of E-cadherin and ZO-1 were quantified by densitometry and normalized with ɑ-tubulin (**i**, **j**) as indicated. Data are means ± SD at least three independent experiments. Means with different superscript letters are significantly different from one another (*P* < 0.05)

Adherent and tight junctions are intercellular junctions crucial for epithelial adhesion and barrier function in the kidney^28^. Since E-cadherin and ZO-1 are two key components responsible for assembling adherent junctions and tight junctions, respectively, we investigated the effect of EZH2 inhibition on the E-cadherin and ZO-1 expression in the injured kidney. Normal expression of E-cadherin and ZO-1 was observed in the sham-operated kidney, but their expression was decreased in the kidneys after IR injury. Interestingly, administration of 3-DZNep largely preserved the levels of E-cadherin and ZO-1 in the injured kidney (Fig. 5a, b). In addition, we also observed decreased expression of E-cadherin and ZO-1 in IR-injured kidneys by immunofluorescence staining. As expected, E-cadherin and ZO-1 expression are limited to renal tubular cells. The E-cadherin and ZO-1 signals were clearly observed in the sham-operated kidney, whereas, their expression was significantly reduced in some tubules of the injured kidneys, but well preserved following 3-DZNep treatment (Fig. 5c–f).

To confirm these observations, we examined the effect of EZH2 inhibition on the expression of E-cadherin and ZO-1 in cultured TKPT injured by H_2_O_2_ by using both 3-DZNep and siRNA approaches. As shown in Fig. 5g, i, EZH2 expression increased with H_2_O_2_ exposure, which was accompanied by reduced expressions of E-cadherin and ZO-1, whereas, 3-DZNep treatment preserved expressions of both. This suggests an important role of EZH2 in mediating disruption of TKPT cell junctions. Similarly, knockdown of EZH2 via its specific siRNA also led to the preservation of E-cadherin and ZO-1 in cultured TKPT injured by H_2_O_2_ (Fig. 5h, j). Of note, inhibition of EZH2 by either 3-DZNep or siRNA also slightly increased the expression of E-cadherin and ZO-1 in TKPT without H_2_O_2_ treatment (Fig. 5g–j), suggesting that the basal level of EZH2 activation plays a role in halting development and maturation of adherent and tight junctions in renal epithelial cells.

### EZH2 inhibition suppresses upregulation of MMP-9 and MMP-2 and downregulation of TIMP-2 and TIMP-3 in the kidney after IR injury and in cultured kidney proximal tubular cells following oxidant injury

**Fig. 6:**
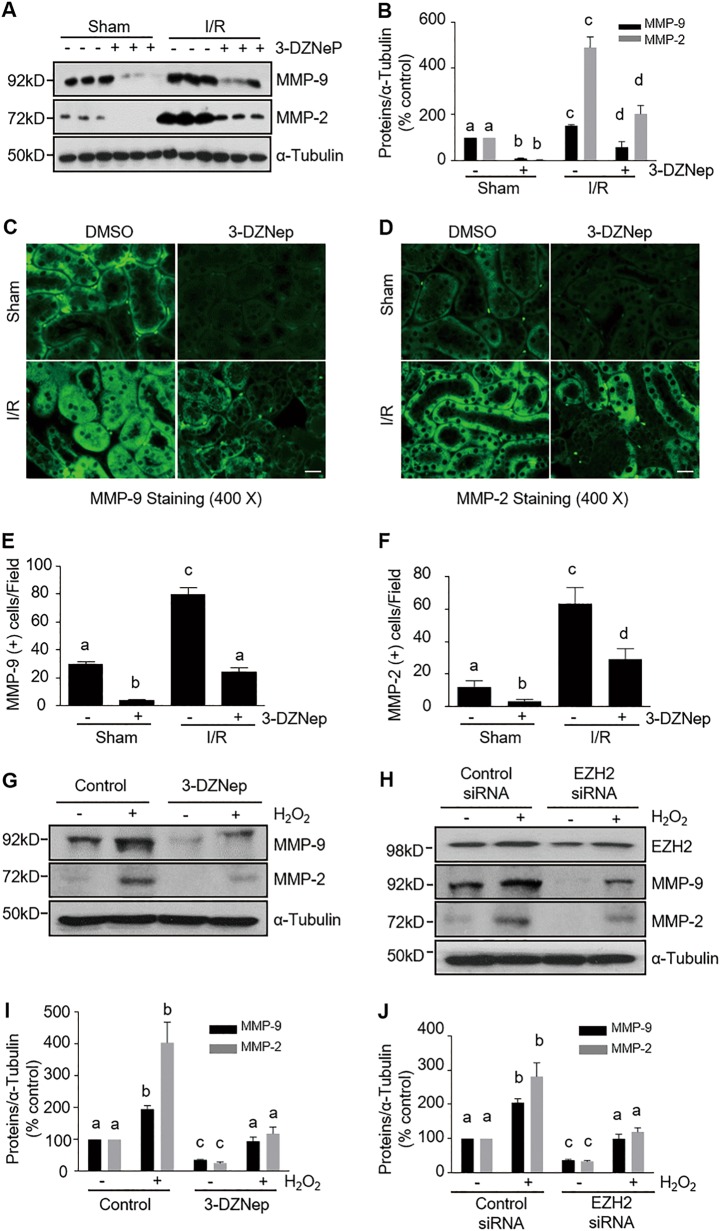
EZH2 inhibition attenuates expression of MMPs in the kidney of IR-induced AKI and H_2_O_2_-injured TKPT. **a** Kidney tissue lysates were subjected to immunoblot analysis with specific antibodies against MMP-9, MMP-2, and ɑ-tubulin. **b** Expression levels of MMP-9 and MMP-2 were quantified by densitometry and normalized with ɑ-tubulin. Data are means ± SD. Representative immunoblots are three samples in each group. **c**, **d** Photomicrographs illustrate immunofluorescent staining of MMP-9 and MMP-2 in kidney tissues collected at 48 h after sham and IR injuring with or without 3-DZNep administration in C57/black mice. Scale bar, 20 µm. **e**, **f** Tubular cells with positive MMP-9 or MMP-2 staining were counted in 10 high-power fields and expressed as means ± SD. TKPT were treated as indicated in Fig. 5. Cell lysates were subjected to immunoblot analysis with specific antibodies against MMP-9, MMP-2 and ɑ-tubulin (**g**, **h**), and expression levels of MMP-9 and MMP-2 (**i**, **j**) were quantified by densitometry and normalized with ɑ-tubulin. Data are means ± SD at least three independent experiments. Means with different superscript letters are significantly different from one another (*P* < 0.05)

**Fig. 7:**
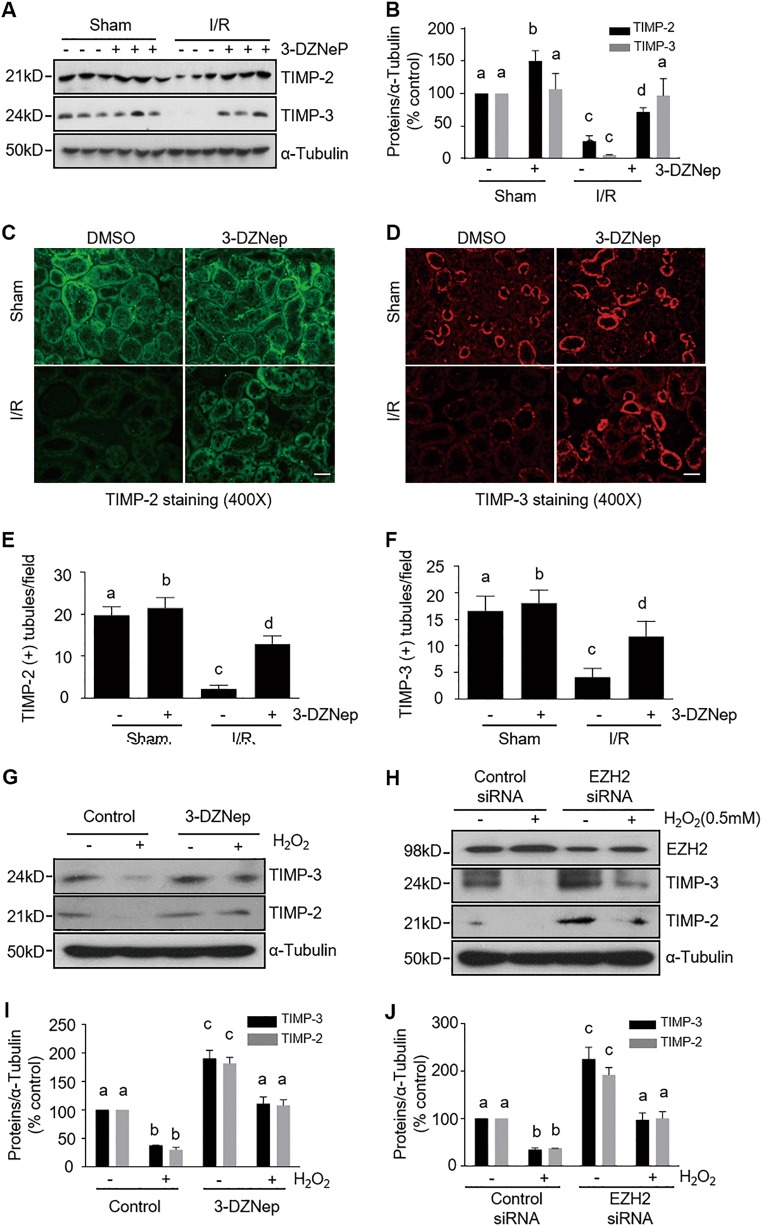
EZH2 inhibition preserves TIMPs expressions in the kidney of IR-induced AKI and H_2_O_2_-injured TKPT. **a** Kidney tissue lysates were subjected to immunoblot analysis with specific antibodies against TIMP-2, TIMP-3 and ɑ-tubulin. **b** Expression levels of TIMP-2 and TIMP-3 were quantified by densitometry and normalized with ɑ-tubulin. Data are means ± SD. Representative immunoblots are three samples in each group. **c**, **d** Photomicrographs illustrate immunofluorescent staining of TIMP-2 and TIMP-3 in kidney tissues collected at 48 h after sham and IR injuring with or without 3-DZNep administration in C57/black mice. Scale bar, 20 µm. **e**, **f** Tubules with positive TIMP-2 and TIMP-3 staining were counted in 10 high-power fields and expressed as means ± SD. TKPT were treated as indicated in Fig. 5. Cell lysates were subjected to immunoblot analysis with specific antibodies against TIMP-2, TIMP-3, and ɑ-tubulin (**g**, **h**), and expression levels of TIMP-3 and TIMP-2 (**i**, **j**) were quantified by densitometry and normalized with ɑ-tubulin. Data are means ± SD at least three independent experiments. Means with different superscript letters are significantly different from one another (*P* < 0.05)

Recent studies show that MMP-2 and MMP-9 have a deleterious effect in IR- induced AKI^29^. Since EZH2 activation plays a key role in suppressing the expression of TIMPs, the negative regulator of MMPs^30^, we examined the role of EZH2 in the regulation of MMP-9, MMP-2, TIMP-2, and TIMP-3 in the kidneys of mice exposed to IR. As shown in Figs. 6a, b,  7a, b, MMP-2, MMP-9, TIMP-2, and TIMP-3 expression was observed in the sham-operated kidneys. However, IR injury to the kidney led to increased expression of MMP-9 and MMP-2, accompanied by decreased expression of TIMP-2 and TIMP-3. In contrast, 3-DZNep administration significantly decreased MMP-9 and MMP-2 expression levels and increased the expression of TIMP-2 and TIMP-3 in IR-injured kidneys. Immunofluorescence staining showed that MMP-9 and MMP-2 were primarily distributed in the cytosol of renal tubular cells; IR-injured kidneys displayed increased density of MMP-9 and MMP-2 signals. 3-DZNep treatment also reduced their expressions in IR-injured and sham-operated kidneys (Fig. 6c, f). In comparison, immunofluorescence staining for TIMPs showed that TIMP-2 and TIMP-3 were expressed in the sham-operated kidney, and their expression levels were reduced in the kidney after IR injury. Treatment with 3-DZNep preserved their expression (Fig. 7c–f). Notably, TIMP-2 and TIMP-3 were expressed in most of the tubular segments, which is consistent with previous observations^31^. These data suggest that EZH2 inhibition may stabilize MMP-9 and MMP-2 activation by preserving TIMP-3 and TIMP-2 levels in the injured kidney, contributing to preserved renal structure and improved function following IR injury.

We further investigated the role of EZH2 in the regulation of MMP-9, MMP-2, and TIMP-3 and TIMP-2 expressions in oxidant injured TKPT. In line with our in vivo observations in the kidney, basal levels of MMP-9, MMP-2, TIMP-3, and TIMP-2 were detected in cultured TKPT cells. Exposure to H_2_O_2_ led to increased expression of MMP-9 and MMP-2 and decreased expression of TIMP-3 and TIMP-2. Interestingly, these reciprocal results were reversed in the presence of 3-DZNep (Figs. 6g, i, 7g, i), or after transfection of siRNA specifically targeting EZH2 (Figs. 6h, j, 7h, j). Therefore, these results suggest that EZH2 activation is able to regulate the balance of MMPs and TIMPs.

### EZH2 inhibition preserves RKIP expression and subsequent inhibition of activation of the Raf/ERK signaling pathway in the kidney following IR injury and in cultured kidney proximal tubular cells following oxidant injury

**Fig. 8:**
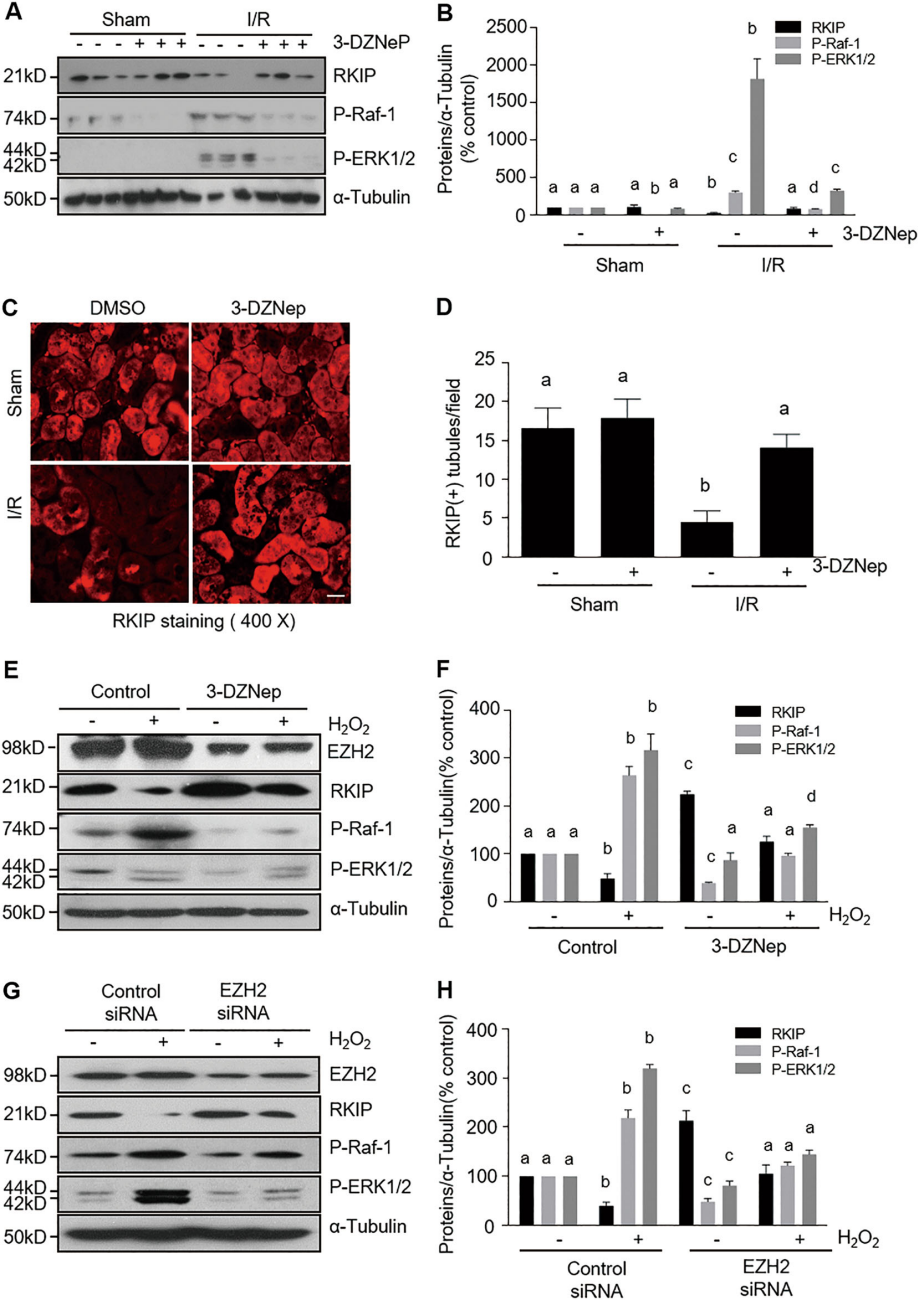
EZH2 inhibition preserves RKIP expression and inhibits phosphorylation of Raf-1 and ERK1/2 in the kidneys of IR-induced AKI or H_2_O_2_-injured TKPT. **a** Kidney tissue lysates were subjected to immunoblot analysis with specific antibodies against RKIP, p-Raf-1, p-ERK1/2 and (**b**) ɑ-tubulin and expression levels of RKIP, p-Raf-1, and p-ERK1/2 were quantified by densitometry and normalized with ɑ-tubulin. Data are means ± SD. Representative immunoblots are three samples in each group. **c** Photomicrographs illustrate immunofluorescent staining of RKIP in kidney tissues collected at 48 h after sham and IR injuring with or without 3-DZNep administration in C57/black mice. Scale bar, 20 µm. **d** Tubules with positive RKIP staining were counted in 10 high-power fields and expressed as means ± SD. TKPT were treated as indicated in Fig. 5. **e**, **g** TKPT cells lysates were subjected to immunoblot analysis with specific antibodies against RKIP, P-Raf-1, P-ERK1/2, and ɑ-tubulin. **f**, **h** Expression levels of RKIP, P-Raf-1, and P-ERK1/2 were quantified by densitometry and normalized with ɑ-tubulin. Data are means ± SD at least three independent experiments. Means with different superscript letters are significantly different from one another (*P* < 0.05)

Previous studies have shown that ERK1/2 activation contributes to both apoptosis and necrosis in renal tubular cells following AKI^26,32^. ERK1/2 is directly activated by MEK1/2, the kinases downstream of Raf-1. Since Raf-1 can be inhibited by RKIP and RKIP is a target of EZH2^18,33^, it is possible that RKIP and ERK1/2 are involved in EZH2-mediated renal tubular cell death. As such, we examined the role of EZH2 in regulating RKIP expression and phosphorylation of Raf-1 and ERK1/2 in vivo and in vitro. The basal level of RKIP was detected by immunoblot analysis in the sham-operated kidney, but the RKIP level was found to be dramatically decreased in the kidney injured by IR. 3-DZNep treatment entirely preserved its expression levels (Fig. 8a, b). In contrast, the phosphorylated (active) forms of Raf-1 (p-Raf-1) and ERK1/2 (p-ERK1/2) were minimally detectable in the sham-operated kidneys, whereas their expression levels were increased in IR-injured kidneys; and administration of 3-DZNep inhibited Raf-1 and ERK1/2 phosphorylation (Fig. 8a, b). Consistent with our immunoblot results, immunofluorescence staining demonstrated equal expression levels of RKIP in almost tubules in the sham-operated kidney, however, expression levels were significantly reduced in IR-injured kidneys and well preserved following 3-DZNep treatment (Fig. 8c, d).

We also investigated the effect of EZH2 inhibition on the expression or phosphorylation of these molecules in H_2_O_2_-injured TKPT cells. Similar to the results found in vivo, RKIP expression levels were decreased after oxidant injury, but remained at control levels when in the presence of 3-DZNep treatment or following transfection with EZH2 siRNA (Fig. 8e–h). On the contrary, H_2_O_2_ exposure led to an increase in the phosphorylation of Raf-1 and ERK1/2, whereas, 3-DZNep treatment or EZH2 siRNA transfection blocked this response (Fig. 8e–h). It should be noted that 3-DZNep treatment or EZH2 siRNA transfection also increased basal levels of RKIP and reduced the basal levels of Raf-1 phosphorylation.

Taken together, these data suggest that inhibition of EZH2 may prevent the activation of the Raf-1/MEK1/2/ERK1/2 signaling pathway and subsequent renal tubular cell death through a mechanism involved in the preservation of RKIP expression.

## Discussion

The purpose of this study was to investigate the role of EZH2 in AKI. Our results reveal that inhibition of EZH2 by 3-DZNep improves renal function and decreases renal tubule damage in murine models of AKI induced by both IR and FA. Inhibiting EZH2 with either 3-DZNep or its specific siRNA also promotes survival of renal epithelial cells in culture following oxidant injury. Moreover, we demonstrated that blocking EZH2 increases expression of its multiple target molecules, including E-cadherin, TIMP-2/3, and RKIP, all of which are associated with inhibition of renal tubular injury. Thus, these results provide strong evidence that EZH2 activation contributes to AKI and suggest that pharmacological targeting of EZH2 might be a novel treatment for patients with AKI.

To our knowledge, this is the first report to demonstrate EZH2 as a critical mediator of AKI. However, it remains unclear how EZH2 contributes to renal tubular cell death and AKI. EZH2 is a histone methyltransferase that induces activity primarily by trimethylating histone H3 at lysine 27 (H3K27me3), subsequently leading to gene suppression. We assumed, therefore, that EZH2 would regulate cell death through epigenetic silencing of genes associated with cell survival, such as E-cadherin^34^, TIMP-2/3^35^, and RKIP^18^. E-cadherin is a transmembrane receptor that mediates cell–cell adhesion and is critical for maintaining epithelial cellular attachment and integrity. Its loss at epithelial junctions can lead to anoikis^36,37^, a form of apoptosis induced by loss of cell–matrix interactions. Consistent with restoration of EZH2 in the kidney after IR injury or in cultured renal proximal tubular cells following oxidant injury, E-cadherin expression levels were found to be remarkably suppressed, whereas blocking EZH2 with 3-DZNep and/or knocking down EZH2 expression with siRNA (in vitro) preserved E-cadherin expression levels. These data strongly suggest that EZH2 activation is able to repress E-cadherin expression. In line with our observations, silencing of EZH2 with short-hairpin EZH2 or 3-DZNep also up-regulated the expression of E-cadherin in cultured renal tumor cells^38^. However, EZH2-mediated regulation of intercellular protein expression is not limited to E-cadherin. In this study, we also observed that EZH2 inhibition preserved the expression of ZO-1, a key molecule involved in the assembly of tight junctions, under conditions of both I/R and oxidant injury. Impairment of both tight junctions and cell–cell adhesion along the lateral membrane of proximal tubule cells provides a paracellular pathway for back leakage of filtrate in renal dysfunction of AKI^39^. EZH2 inhibition resulting in preservation of adherent/tight junction proteins (i.e. E-cadherin and ZO-1) may be a critical mechanism for maintaining the integrity of epithelial junctional complexes, preventing epithelial cell detachment and death, and protecting against AKI.

TIMPs are endogenous inhibitors of MMPs, and silencing of TIMPs, in particular, TIMP-3, has been reported to increase the activity of multiple MMPs, disrupt the basal membrane, and interfere with cell–matrix adhesion^40^, eventually leading to cell death. Several studies have demonstrated that MMP-9 and MMP-2 are upregulated after I/R^22,41^, and that increased MMP-2 and MMP-9 expression and activation are associated with acute tubular injury and increased microvascular permeability following I/R^6,41^. It has been reported that EZH2 can attenuate expression of TIMP-2 and TIMP-3 by inducing gene promoter DNA methylation, thereby increasing MMP activity in tumor cells^30,35^. In the current study, we found that injury to the kidney or cultured renal epithelial cells resulted in decreased expression of TIMP-2 and TIMP-3 and reciprocally increased expressions of MMP-2 and MMP-9, whereas, inhibition of EZH2 blocked these responses. This suggests that EZH2 may mediate injury-induced transcriptional repression of the TIMP genes and subsequent upregulation of MMP-2 and MMP-9. As such, blocking EZH2 would shift the MMPs/TIMPs balance in favor of suppression of MMP activity and thus promote cell survival.

As a potent inhibitor of Raf-1 kinase, RKIP exhibits a strong endogenous inhibitory effect on the Raf/MEK/ ERK signaling pathway^42–44^. Previously, we have shown that activation of the ERK1/2 pathway leads to both renal tubular cell apoptosis and necrosis in response to oxidative stress in primary culture^25^. Other studies have confirmed the role of ERK1/2 signaling in mediating I/R and cisplatin-induced AKI in animal models. Since EZH2 has been reported to negatively regulate RKIP transcription through repression-associated histone modifications^18^, we also examined whether injury to the kidney would alter RKIP expression and/or whether EZH2 plays a role in this process. Our data showed that RKIP was abundantly expressed in the renal tubular cells in vitro and in vivo; and renal IR injury reduced its expression, which was inversely correlated with phosphorylation of Raf-1 and ERK1/2. Interestingly, this response was totally blocked by inhibition of EZH2 with 3-DZNep. In parallel, siRNA-mediated silencing of EZH2, or 3-DZNep treatment, also promoted cell survival in cultured renal epithelial cells following oxidant injury, which corresponds with the recovery of the lost RKIP expression and inhibition of Raf-1 and ERK1/2 phosphorylation. Hence, EZH2 may contribute to renal epithelial cell death through a mechanism associated with epigenetic repression of RKIP and subsequent activation of the Raf-1/MEK/ERK signaling pathway. However, we cannot rule out the possibility that EZH2 mediates the death of renal tubular cells through regulating expression/activation of other RKIP-regulated signaling molecules. In this aspect, RKIP has been reported to be a repressor of NF-κ B, a well-identified transcriptional factor that contributes to cell survival^45^. Further investigations must be undertaken to elucidate the role of EZH2 in regulating activation of NF-κB signaling in the kidney after acute injury.

Accumulating evidence strongly suggests that EZH2 expression levels/activity are up-regulated in cancers. Several EZH2 inhibitors including 3-DZNep are currently being tested in pre-clinical models, and other inhibitors, such as GSK126, EPZ-6438, CPI-1205 are under clinical trials as anti-cancer drugs^14^. By utilizing 3-DZNep, we demonstrated that blocking EZH2 also protects against IR-induced and FA-induced AKI in mice and inhibits renal tubular cell injury in culture. The renoprotective effects of EZH2 inhibition involve the attenuation of renal tubular cell death through the targeting of multiple cellular events. These include the preservation of adherent/tight junction proteins, maintenance of the MMP/TIMP balance, and inhibition of the ERK pathway. Given that EZH2 inhibitors have been recently considered as potential epigenetic drugs, pharmacologic targeting of EZH2 could be a novel approach for treatment of AKI.

## Materials and methods

### Antibodies and reagents

Antibodies to EZH2, H3K27me3, RKIP, cleaved Caspase-3, and P-ERK1/2 were purchased from Cell Signaling Technology (Danvers, MA). Antibodies to E-cadherin, MMP-2, P-Raf-1, and GAPDH were obtained from Santa Cruz Biotechnology (Santa Cruz, CA). Cleaved PARP (24 kDA) and TIMP-2 antibodies were purchased from Abcam, Inc. (Cambridge, MA). Neutrophil gelatinase-associated lipocalin NGAL antibody was obtained from R&D Systems (Minneapolis, MN). ZO-1, MMP-9, TIMP-3 antibodies were purchased from EMD Millipore (Billerica, MA). 3-DZNeP was purchased from Selleck Chemicals (Houston, TX). ɑ-tubulin and all other chemicals were purchased from Sigma-Aldrich (St. Louis, MO). SiRNA specific for EZH2 was obtained from Invitrogen (Carlsbad, CA). All other antibodies used in this study were purchased from Cell Signaling Technology (Danvers, MA).

### Animals and treatment

Male C57/black mice, each weighing 20–25 g (Jackson Laboratory, Bar Harbor, ME), were housed under a 12:12 h light–dark cycle with food and water supplied ad libitum. The I/R model was established in 6-week-old to 8-week-old male C57/black mice, with six mice in each group. Mice were anesthetized with 75 mg/kg ketamine (i.p.) and 50 mg/kg dexmedetomidine (Dexdomitor) (i.m.). The renal arteries and veins were isolated from the surrounding tissue by blunt dissection and then occluded with a non-traumatic vascular clamp for 30 min at 37 °C. The sham-operated kidney was used as the control, in which the renal pedicles were isolated, but no clamps were applied. To establish FA-induced AKI, the animals were injected intraperitoneally with FA at 250 mg/kg body weight. Sodium bicarbonate (0.3 M NaHCO_3_, the vehicle used for FA administration) alone was used as controls. To examine the efficacy of EZH2 inhibition on AKI, 3-DZNep (2 mg/kg) in 50 μl of DMSO was given by i.p. immediately following IR injury or FA injection and then administered daily until mice were sacrificed. Animals treated with an equal volume of DMSO were used as controls. At 24 or 48 h after surgery, animals were sacrificed and the kidneys were removed for protein analysis and histological examination.

### Measurement of renal function

Renal function was estimated by serum creatinine and blood urea nitrogen (BUN), measured using a colorimetric kit (Sigma Diagnostics) and enzymatic assay Kit (Sigma Diagnostics), respectively, according to the protocol provided by the manufacturer.

### Assessment of tubular injury

A TUNEL staining kit was used to detect DNA strand breaks according to the instructions provided by Roche Molecular System (Branchburg, NJ). The number of TUNEL-positive nuclei per field (*n* = 10) was evaluated.

### Cell culture and treatment

Murine proximal tubule epithelial (TKPT) cells were cultured in DMEM/Nutrient F12 (DMEM/F12) containing 5% fetal bovine serum (FBS) and 0.5% penicillin and streptomycin in an atmosphere of 5% CO_2_–95% ambient air at 37 °C. TKPT cells were 60–70% confluent when used for various treatments. To determine the effect of EZH2 inhibition on H_2_O_2_-induced TKPT cell injury, TKPT cells were starved for 24 h with DMEM/F12 containing 0% FBS and then, treated with 3-DZNep or water for 1 h and additionally exposed to H_2_O_2_ (0.5 mM) for 12 h.

### Transfection of siRNA into cells

In a six-well plate, TKPT cells were seeded to 50–60% confluence in FBS and antibiotic-free medium and grown for 24 h. Then, cells in each well were transfected with siRNA (100 pmol) specific for EZH2 using Lipofectamine 2000 (Invitrogen, Carlsbad, CA) according to the manufacturer’s instructions. In parallel, scrambled siRNA (100 pmol) was used as a control for off-target changes in TKPT cells; 24 h after transfection, the medium was changed, and cells were incubated with H_2_O_2_ (0.5 mM) for an additional 12 h before being harvested for analysis.

### Immunoblot analyses

Kidney tissue samples or cultured cells were homogenized with cell lysis buffer and a protease inhibitor cocktail. Proteins were separated by SDS–PAGE and transferred to nitrocellulose membranes. After incubation with 5% non-fat milk for 1 h at room temperature, membranes were incubated with a primary antibody overnight at 4 °C and then exposed to appropriate horseradish peroxidase-conjugated secondary antibodies for 1 h at room temperature. Bound antibodies were visualized by chemiluminescence detection.

### Histochemical and immunofluorescence staining

Tissues were fixed in 4.5% buffered formalin, dehydrated, and embedded in paraffin. Sections were stained with hematoxylin and eosin (HE). For immunofluorescent staining, primary antibodies against EZH2 (1:200), NGAL (1:200), E-cadherin (1:50), ZO-1(1:200), MMP-9 (1:200), MMP-2 (1:50), TIMP-3 (1:200), TIMP-2 (1:200), and RKIP (1:200) and fluorescent-conjugated secondary antibodies (1:300) were applied to the sections. Examination and scoring of sections from each kidney (*n* = 10 for each condition) were carried out in a blinded fashion. Tubular injury was scored on a scale from 0 to 3, where 0 = normal, 1 = injury <30%, 2 = injury 30–60%, 3 = injury >60%.

### Densitometry analyses

The semi-quantitative analysis of different proteins was carried out using ImageJ software developed at the National Institutes of Health. Quantification is based on the intensity (density) of the band, which is calculated by the area and pixel value of the band. Quantification data are given as a ratio between target protein and loading control (housekeeping protein).

### Statistical analyses

Data are presented as means ± SD and were subjected to one-way ANOVA. Multiple means were compared using Tukey test, and differences between two groups were determined by *t* test. *P* < 0.05 was considered statistically significant.

## Electronic supplementary material

Supplemental materials
