# Supplementary material for: Targeting enhancer of zeste homolog 2 protects against acute kidney injury
Source: Cell Death Dis. 2018 Oct 19;9(11):1067. doi: 10.1038/s41419-018-1012-0 (PMC6195522; doi:10.1038/s41419-018-1012-0)
Supplement: Supplementary file 1 — Supplemental materials [file 41419_2018_1012_MOESM1_ESM.docx]

**Supplemental Documents**

**Targeting Enhancer of Zeste Homolog 2** **Protects Against Acute Kidney Injury**

Xiaoxu Zhou, Xiujuan Zang, Yinjie Guan, [Evelyn Tolbert](http://www.ncbi.nlm.nih.gov/pubmed/?term=Tolbert%2520E%255Bauth%255D), [Ting C. Zhao](http://www.ncbi.nlm.nih.gov/pubmed/?term=Zhao%2520TC%255Bauth%255D), George Bayliss, and Shougang Zhuang

**
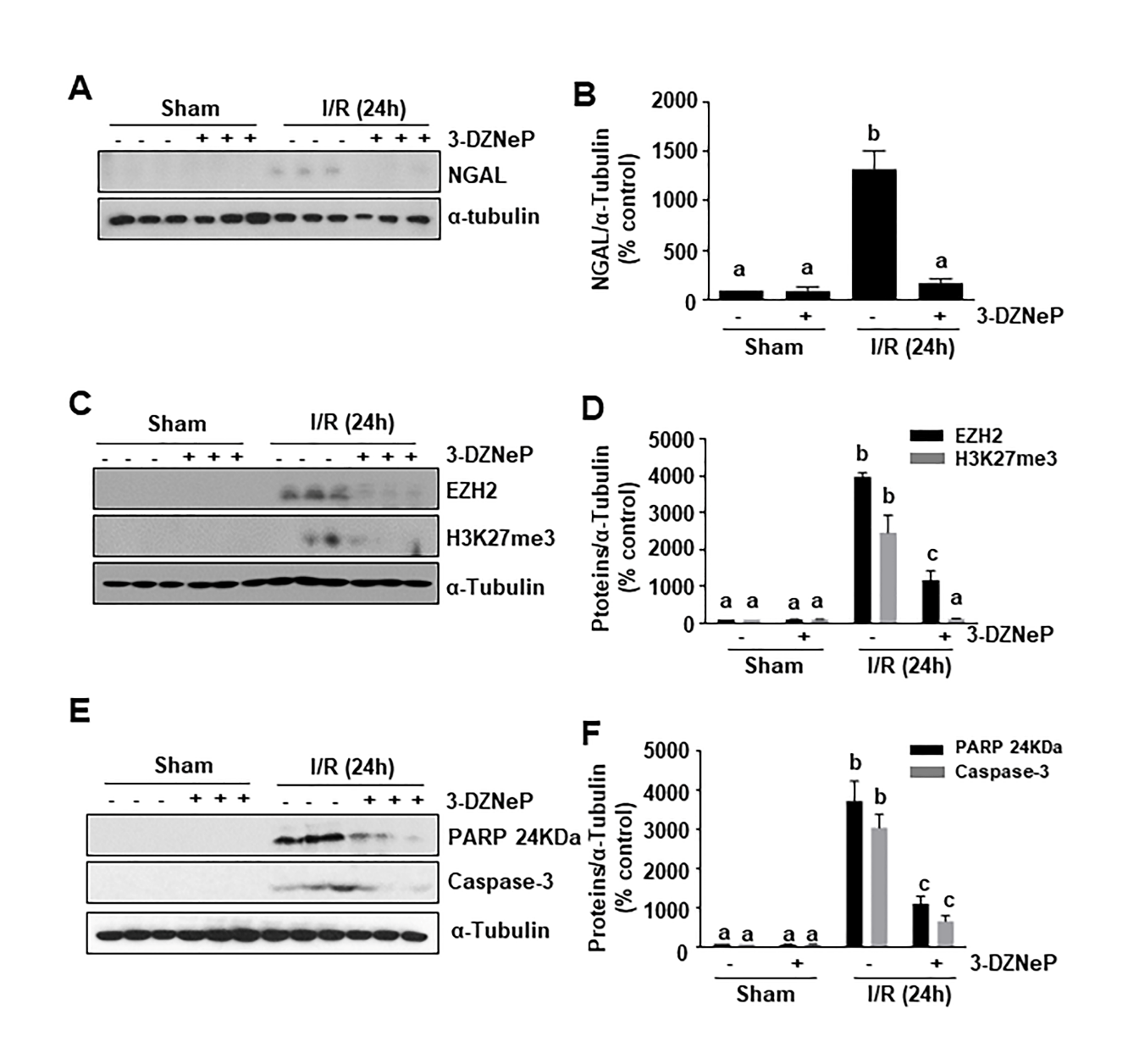
Figure S1.** **Enhancer of Zeste Homolog 2** (**EZH2) inhibition inhibits renal tubular cell injury and death in the kidneys of ischemia/reperfusion (I/R)--induced acute kidney injury (AKI).** The kidneys of C57/black mice were subjected to ischemia as indicated in Materials and Methods and then reflowed for 24 hours with or without 3-Deazaneplanocin A (3-DZNep) administration. Tissue lysates were subjected to immunoblot analysis with specific antibodies against neutrophil gelatinase-associated lipocalin (NGAL) (A), EZH2, histone H3 lysine 27 trimethylation (H3K27me3) (C), cleaved poly (ADP-ribose) polymerase PARP (24 kDa) and cleaved Caspase-3 (E) and ɑ-tubulin (A,C, E). Expression levels of NGAL (B), EZH2, H3K27me3 (D) cleaved PARP or cleaved Caspase-3 (F) were quantified by densitometry, normalized with ɑ-tubulin and expressed as percentage of controls. Means with different superscript letters are significantly different from one another (P < 0.05).

**Figure S2. Enhancer of Zeste Homolog 2** (**EZH2) inhibition reduces death of cultured kidney proximal tubular epithelial (**TKPT) **cells in response to oxidant injury.** TKPT were pretreated with various concentration of 3-Deazaneplanocin A (3-DZNep) (1-5 μM) for 1 hour (G) or transfected with control or EZH2 siRNA for 24 hours (H) and then exposed to 0.5 mM H_2_O_2_ for an additional 12 hours, cell survival was examined by the MTT assay. Data are means ± SD from at least three individual experiments. Means with different superscript letters are significantly different from one another (P < 0.05).
